# Novel ideas for the comprehensive evaluation of varus knee osteoarthritis: radiological measurements of the morphology of the lateral knee joint

**DOI:** 10.1186/s13018-023-03684-x

**Published:** 2023-03-13

**Authors:** Wenru Ma, Fengkun Wang, Shengnan Sun, Lei Ding, Lu Wang, Tengbo Yu, Yi Zhang

**Affiliations:** 1 Department of Sports Medicine, Affiliated Hospital of Qingdao University, 59 Haier Road, Laoshan District, QingDao, 266000 Shandong Province China; 2 Department of Clinical Medicine, Qingdao University, Qingdao, Shandong Province China; 3 Department of Quality Management Evaluation, Affiliated Hospital of Qingdao University, Qingdao, Shandong Province China; 4 Department of Education and Training, Affiliated Hospital of Qingdao University, Qingdao, Shandong Province China; 5 Department of Orthopedics, Affiliated Hospital of Qingdao University, Qingdao, Shandong Province China; 6 Institute of Sports Medicine and Rehabilitation, Qingdao University, Qingdao, Shandong Province China; 7 Shandong Institute of Traumatic Orthopedics, Qingdao, Shandong Province China

**Keywords:** Varus knee osteoarthritis, Genu varus, Radiology, Lateral plateau widening, Proximal fibula curvature, Fibula height

## Abstract

**Background:**

The lateral anatomical and morphological characteristics of knees with varus knee osteoarthritis (OA) have not received sufficient attention. This study used several radiological parameters to describe the morphological characteristics of the lateral knee with OA to determine whether there are relationships between varus knee OA and parameters such as lateral plateau widening (LPW), proximal fibula curvature (PFC), and fibula height (FH).

**Methods:**

The study retrospectively analyzed 1072 subjects who underwent standard radiography for diagnosing or evaluating symptomatic knee joint disease. The 163 Kellgren and Lawrence (K–L) grades 0 and I knees were categorized into the no-knee-OA group, and the 909 K–L grades II–IV knees were classified into the knee-OA group. Medial proximal tibial angle, joint line convergence angle, hip–knee–ankle angle, LPW, PFC, and FH were measured. T tests and chi-square tests were used to compare each index between the two groups. Binary logistic regression was performed to examine the correlation between indexes and knee OA occurrence. Ordinal logistic analysis, principal component analysis, and multivariable linear regression analysis were performed to examine the correlations between the three lateral parameters and K–L grades and the degree of varus deformity.

**Results:**

LPW and PFC were significantly greater and FH was significantly smaller in the knee-OA group than in the no-knee-OA group. LPW, PFC, and FH were correlated with knee OA occurrence. One principal component, named the comprehensive principal component score of varus deformity, was extracted from the three indexes, and the total variance of the principal component interpretation was 76.60%. Ordinal logistics and multivariable linear regression analysis showed that, after adjusting for age and BMI, LPW and PFC were positively correlated with K–L grading and varus deformity. FH was significantly and negatively correlated with K–L grading and varus deformity (all *P* < 0.05).

**Conclusions:**

Regular morphological changes take place in the lateral knee with varus OA, including lateral dislocation of the tibial plateau, proximal fibula bending, and upward movement of the fibular head. Changes in LPW, PFC, and FH could enable a more comprehensive assessment of varus knee OA occurrence, severity, and deformity.

**Level of evidence** Retrospective Study Level III.

## Background

Knee osteoarthritis (OA) is the main cause of chronic pain and disability in middle-aged and older adults and is treated with knee arthroplasty or high tibial osteotomy [1–4]. Varus knee OA caused by varus knee deformity is more common than other OA subtypes [5]; this is the result of ongoing elevated pressure in the medial knee compartment [6].

Radiography is important for diagnosing and evaluating knee diseases. The Kellgren and Lawrence (K–L) grading system is commonly used to evaluate knee joint degeneration based on radiographic findings. The medial proximal tibial angle (MPTA), joint line convergence angle (JLCA), and hip–knee–ankle angle (HKAA) are indexes that are commonly used to assess the degree of lower limb varus deformity.

However, there is little systematic research on the lateral knee with varus OA and few parameters for evaluating degeneration of the lateral knee structures, such as the lateral femoral condyle, lateral tibial plateau, and proximal fibula. Radiological changes in these structures are observed in patients with severe varus knee OA. First, the lateral joint lines of the tibia and femur are misaligned. According to Johannsen et al., lateral plateau widening is useful for evaluating lateral tibial plateau fractures and provides information on how to assess lateral alignment in knee OA [7]. Second, the proximal fibula becomes more curved. Weight-bearing by the fibula, which alters the mechanical distribution of the medial and lateral knee compartment loads, is associated with the pathogenesis of knee OA [8, 9]. Just as a phenomenon, fibula bending has been documented in varus knee OA [10]. Still, there are no comprehensive studies of its relationship with the severity of knee OA or adaptive tibial changes. Its possible role in the assessment of knee OA has also been ignored. Moreover, the fibula head appears to be closer to the lateral tibial plateau. The fibula shifts downward after a proximal fibula osteotomy, which is a new surgery for treating varus knee OA that leads to symptom relief [9], thus suggesting that there is a connection between fibula height and varus knee OA. Systematic clinical studies are needed to clarify these findings for the comprehensive evaluation of varus knee OA.

Therefore, in this novel study, we proposed to focus on radiological changes in the lateral knee joint with varus OA and examined parameters such as lateral plateau widening (LPW), proximal fibula curvature (PFC), and fibula height (FH). We then investigated the relationship between these parameters and the degeneration and varus deformity of the knee compared with traditional indexes such as K–L grade, MPTA, JLCA, and HKAA.

## Methods

### Study population

From September 2018 to August 2020, 3127 subjects underwent standard anteroposterior (AP), and lateral radiographs and weight-bearing full-leg AP radiographs to evaluate symptomatic knee joint disease in the Department of Sports Medicine, the Affiliated Hospital of Qingdao University. The inclusion criteria were (1) > 45 years of age, (2) no history of knee surgery, and (3) standard AP and lateral and weight-bearing full-leg AP radiography. The exclusion criteria were (1) previous knee trauma or surgery, (2) nondegenerative OA, such as rheumatoid arthritis, Kashin–Beck disease, Dendrolimus OA, hemophilic arthritis, or inflammatory arthritis, (3) congenital deformity or valgus deformity or skeletal dysplasia of the knee, (4) ligament laxity or stiff knee, and (5) tumor or osteopathy of the knee joint. Ultimately, the study enrolled 1072 knees [376 males, 696 females; mean subject age 66.84 (range 46–83) years; mean body mass index (BMI) 26.98 (range 19.43–37.05) kg/m^2^]. Of the subjects, 909 had different degrees of OA, and the other 163 were healthy. The 2055 excluded knees had a valgus deformity, skeletal dysplasia, rheumatoid arthritis, incomplete radiological data, poor image quality, or lower limb trauma or surgery history.

### Radiological measurements

In order to evaluate symptomatic knee joint disease, X-ray examinations, including standard AP and lateral radiographs of the knee and weight-bearing full-leg AP radiographs, were performed in all subjects. The measurements were made independently by one radiologist with 4 years of experience in postprocessing procedures and by a second radiologist with 2 years of experience in orthopedic radiology using the picture archiving and communication system. The patient’s position was adjusted so that the toes were straight ahead, with the feet separated sufficiently for balance, and the patient’s weight equally distributed on the feet. The radiographic severity of knee OA was assessed with the Kellgren and Lawrence (K–L) grading system [11, 12]. Knees of K–L grades 0 and I were categorized as no-knee OA, and knees of K–L grades II–IV were categorized as knee OA [13].

**Fig. 1:**
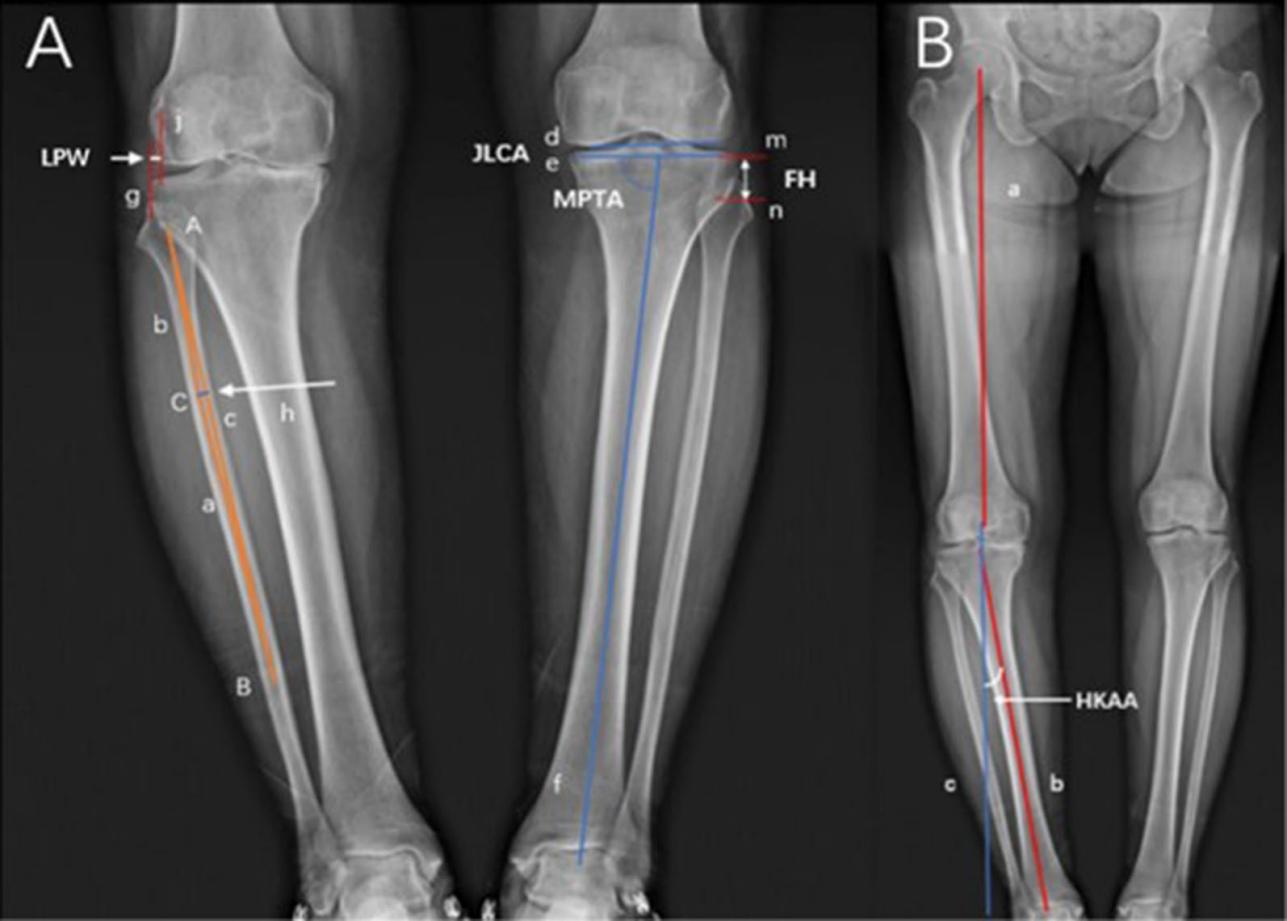
Schematic diagram of the imaging measurement methods. **A** MPTA was measured as the angle between line e and line f; JLCA was measured as the angle between line d and line e; FH was measured as the shortest distance between line m and line n; LPW was measured as the shortest distance between line g (line along the lateral-most aspect of the proximal tibia) and line j (lateral margin of the distal femoral condyle); PFC was measured as the reciprocal of the radius of the proximal fibula circumcircle, lengths of line a, line b, line c, and line h were measured, respectively. **B** HKAA was measured as the angle between the line b and line c, and line c is the extension of line a

Parameters were measured on standard weight-bearing full-leg AP radiographs as shown in Fig. 1. MPTA was measured as the angle between the tibial plateau and the mechanical axis of the tibia. JLCA was measured as the angle between the line connecting the distal femur and proximal tibial articular surfaces on AP radiographs. Intra-articular varus deformity due to the narrowing of the medial joint space often manifests as a positive JLCA value. HKAA was measured as the angle between the mechanical axes of the femur and tibia. Lateral plateau widening (LPW) was measured as the distance between the lateral line along the lateral margin of the distal femoral condyle and the line along the lateral-most aspect of the proximal tibia, both of which are perpendicular to the horizontal plane. Proximal fibula curvature (PFC) was defined as the degree of curvature of the proximal fibula. Fibula is S-shaped and artificially divided into upper, middle, and lower segments. As can be seen in Fig. 1A, point A represents the center of the fibular head (the center point of the line at the widest position of the fibular head), point B represents the distal point of the middle segment of the fibula, and point C is the center point of the fibula shaft where was of the maximum curvature between point A and point B. Vertical line h was from point C to line c. Lengths of line a, line b, line c, and line h were measured, respectively. PFC was measured as the reciprocal of the radius of the proximal fibula circumcircle using the formula below. Fibula height (FH) was measured as the shortest distance from the tibial plateau to the base of the fibular styloid process on plain radiographs.

### Formula for PFC

The radius (r) of the circumcircle of triangle ABC is $\frac{abc}{4S}$ (*S* is the area of triangle ABC). The curvature of the circumcircle is $\frac{1}{r}$ (Fig. 1).

$${\text{So}},{\text{ PFC}} = \frac{1}{r} = \frac{4S}{{abc}} = { }\frac{2h}{{ab}}$$

### Statistical analysis

The Kolmogorov–Smirnov normality test was used before the statistical analyses to determine whether to use a parametric test. Continuous variables conforming to the normal distribution were expressed as the mean and standard deviation, and a *t* test was performed to compare radiological measurements between the no-knee-OA and knee-OA groups. Categorical variables were expressed as frequencies (%), and the groups were compared with the chi-square test. Binary logistic regression was performed to determine independent risk factors for knee OA. Kendall’s tau-b correlation analysis was performed to compare the radiological parameters and K–L grades, and Pearson’s correlation analysis was performed to compare the radiological parameters and MPTA, JLCA, and HKAA. The absolute correlation coefficient (*r*) was used to indicate very strong (*r* = 0.80–1.00), strong (*r* = 0.60–0.79), moderate (*r* = 0.40–0.59), weak (*r* = 0.20–0.39), and no (*r* < 0.20) correlations. If it was also significantly correlated with MPTA, JLCA, and HKAA, principal component analysis was used to calculate the comprehensive principal component score of varus deformity, which was used to comprehensively evaluate the degree of varus knee deformity by reducing the dimensions of the original data. Odds ratios (ORs) were calculated using ordinal logistic regression to determine whether the radiological parameters were associated with K–L grades, and multivariable linear regression was used for comparison with the comprehensive principal component score of varus deformity. Intra- and interclass correlation coefficients (ICCs) with 95% confidence intervals (CIs) were used to assess intra- and interrater variability. ICC > 0.75 was considered to represent excellent agreement. All statistical evaluations were performed using PASW Statistics (25.0, SPSS, Chicago, IL, USA). A *P-*value < 0.05 was considered to be statistically significant.

## Results

### Basic information

A total of 1072 participants were included in this study and underwent standard radiography. A total of 909 subjects suffering from different degrees of OA, and the other 163 were normal and healthy. Good-to-excellent intraobserver and interobserver variability was achieved for all measurements; the data are presented in Table 1.

**Table 1:** Intra- and inter-reliabilities of imaging measurement indexes

	First vs. second assessment by 1 examiner		Assessment by examiner 1 vs. examiner 2	
	ICC	95% CI	ICC	95% CI
MPTA	0.928	0.913–0.940	0.902	0.891–0.919
JLCA	0.920	0.911–0.939	0.878	0.860–0.903
HKAA	0.933	0.916–0.967	0.917	0.899–0.926
LPW	0.925	0.919–0.933	0.873	0.844–0.912
PFC	0.919	0.912–0.928	0.847	0.825–0.880
FH	0.938	0.922–0.953	0.889	0.861–0.920

*MPTA* medial proximal tibial angle, *JLCA* joint line convergence angle, *HKAA* hip–knee–ankle angle, *LPW* lateral plateau widening, *PFC* proximal fibula curvature, *FH* fibular head height, *ICC* intra- or interclass correlation coefficient, *CI* confidence interval

### All indexes comparison between knee OA and no-knee OA

Table 2 shows each index measured and compared between the knee OA (K–L grades II–IV) and no-knee OA groups (K–L grades 0–I). Significant differences were found in age, BMI, MPTA, JLCA, HKAA, LPW, PFC, and FH (all *P* < 0.05) but not in sex (*P* = 0.110) between the two groups.

**Table 2:** Comparison between groups

	Knee OA group	No-knee OA group	*t*/*χ*^2^ value	*P* value
Age	67.82 ± 6.31	61.42 ± 8.35	−8.992	< 0.001
Sex				
Male	300 (0.33)	77 (0.47)	1.626	n.s
Female	609 (0.67)	86 (0.53)		
BMI (kg/m^2^)	27.18 ± 3.27	25.81 ± 2.62	−5.721	< 0.001
LPW (mm)	5.1 ± 2.1	1.9 ± 1.1	−28.562	< 0.001
PFC (10^–5^ × mm^−1^)	16.1 ± 9.9	5.3 ± 2.8	−26.386	< 0.001
FH (mm)	17.5 ± 2.3	21.8 ± 1.6	28.866	< 0.001
MPTA (°)	84.28 ± 2.69	87.41 ± 1.41	21.210	< 0.001
JLCA (°)	5.95 ± 2.63	0.88 ± 0.45	−52.073	< 0.001
HKAA (°)	9.95 ± 4.89	2.51 ± 1.55	−35.439	< 0.001

*OA* osteoarthritis, *BMI* body mass index, *LPW* lateral plateau widening, *PFC* proximal fibula curvature, *FH* fibular head height, *MPTA* medial proximal tibial angle, *JLCA* joint line convergence angle, *HKAA* hip–knee–ankle angle; n.s., *P* > 0.05

### Correlations between lateral parameters and knee OA occurrence

To evaluate the influence of lateral parameters on knee OA occurrence, LPW, PFC, and FH were taken into account. Binary logistic regression analysis showed that after adjusting for age and BMI, significant correlations were found between LPW, PFC, FH, and knee OA occurrence (*P* < 0.05) (Table 3).

**Table 3:** Logistic regression analysis of risk factors for knee OA occurrence

	B	SE	*P* value	OR (95%CI)
Age	0.141	0.024	< 0.001	1.15 (1.10–1.21)
Sex			n.s	
LPW	0.844	0.151	< 0.001	2.33 (1.73–3.13)
PFC	0.308	0.049	< 0.001	1.36 (1.24–1.50)
FH	−0.718	0.099	< 0.001	0.49 (0.40–0.59)

*OA* osteoarthritis, *BMI* body mass index, *LPW* lateral plateau widening, *PFC* proximal fibula curvature, *FH* fibular head height, *B* regression coefficient, *SE* standard error of regression coefficient, *OR* odds ratio, *CI* confidential interval, n.s., *P* > 0.05

### Correlations between lateral parameters and knee OA severity and varus deformity

**Fig. 2:**
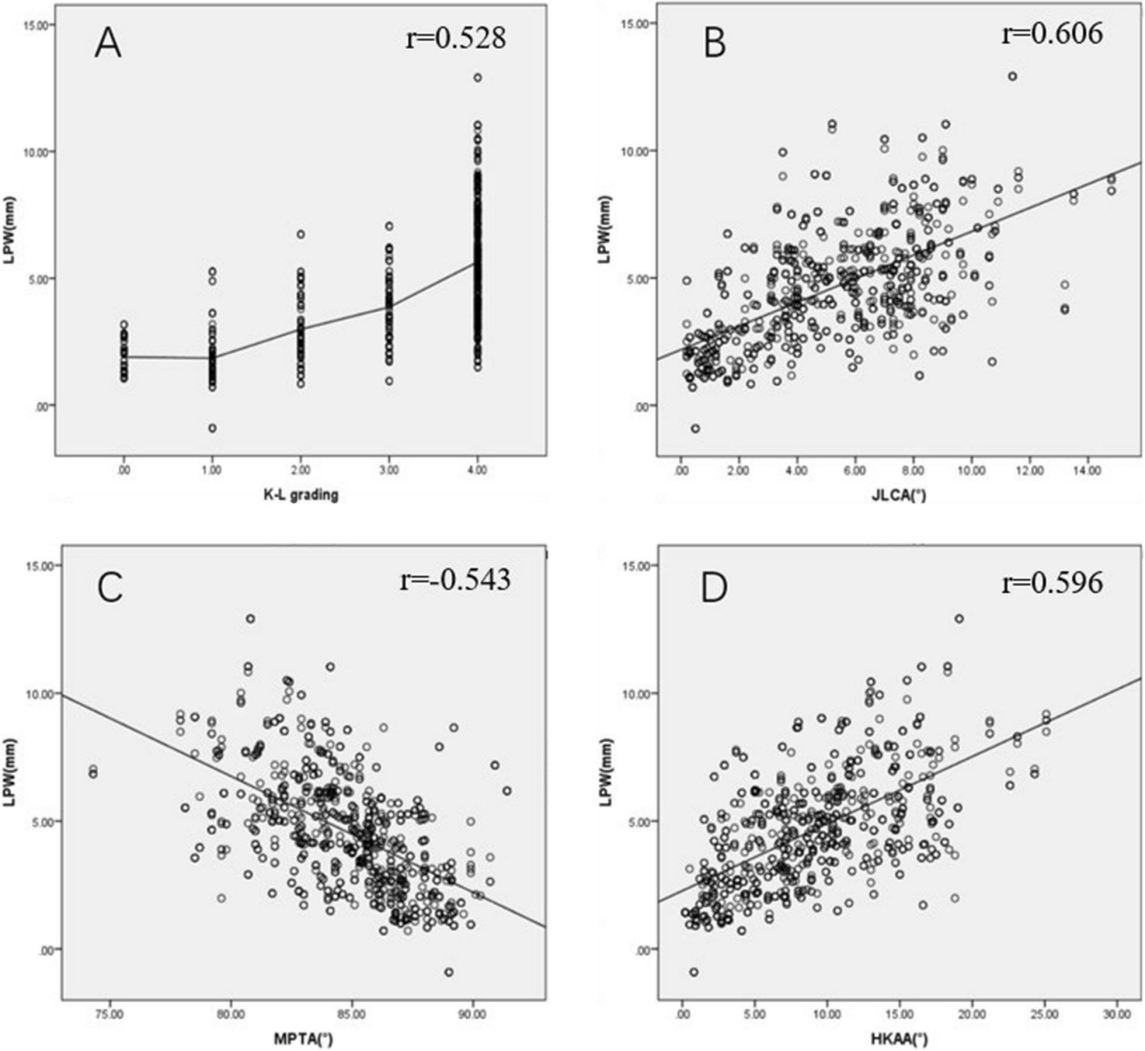
Results of correlation analysis. LPW, lateral plateau widening; K–L grading, Kellgren and Lawrence grading; JLCA, joint line convergence angle; MPTA, medial proximal tibial angle; HKAA, hip–knee–ankle angle.** A** The higher the K–L grade, the bigger the LPW;** B** LPW is positively correlated with JLCA;** C** LPW is negatively correlated with MPTA; ** D** LPW is positively correlated with HKAA. All *P* < 0.001

**Fig. 3:**
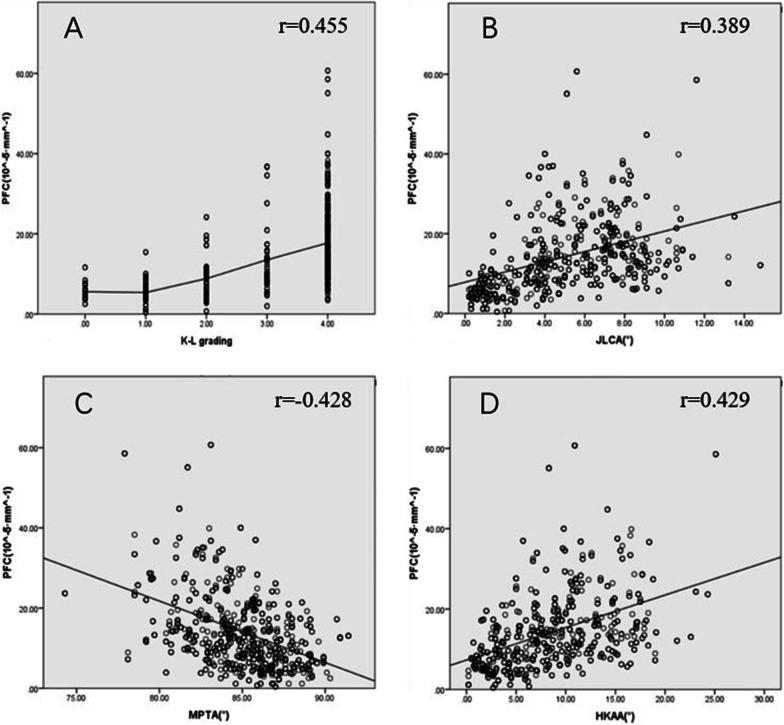
Results of correlation analysis. PFC, proximal fibula curvature; K–L grading, Kellgren and Lawrence grading; JLCA, joint line convergence angle; MPTA, medial proximal tibial angle; HKAA, hip–knee–ankle angle. **A** The higher the K–L grade, the bigger the PFC; **B** PFC is positively correlated with JLCA; **C** PFC is negatively correlated with MPTA; **D** PFC is positively correlated with HKAA. All *P* < 0.001

**Fig. 4:**
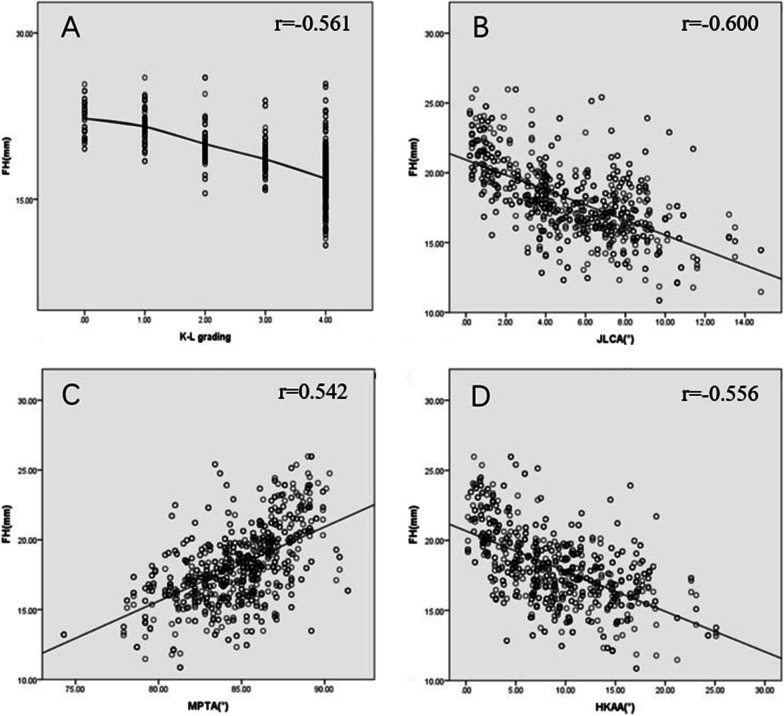
Results of correlation analysis. FH, fibular head height; K–L grading, Kellgren and Lawrence grading; JLCA, joint line convergence angle; MPTA, medial proximal tibial angle; HKAA, hip–knee–ankle angle. **A** The higher the K–L grade, the smaller the FH; **B** FH is negatively correlated with JLCA; **C** FH is positively correlated with MPTA; **D** FH is negatively correlated with HKAA. All *P* < 0.001

Kendall’s tau-b and Pearson’s correlation analysis showed that LPW, PFC, and FH had significant correlations with each conventional OA parameter (*P* < 0.05) (Figs. 2, 3, 4).

Results of correlation analysis among LPW, PFC, and FH showed that there are correlations among the three lateral parameters, with *r* = 0.353 (LPW and PFC), −0.451(LPW and FH), and −0.384 (PFC and FH), respectively, all *P* < 0.001. Pairwise correlations between MPTA, JLCA, and HKAA were significant, with *r* = −0.455 (MPTA and JLCA), −0.737 (MPTA and HKAA), and 0.742 (JLCA and HKAA), respectively, all *P* < 0.001. Therefore, principal component analysis was performed. A total of 1 principal component, named the comprehensive principal component score of varus deformity, was extracted from the three indexes, and the total variance of the principal component interpretation was 76.60%, indicating adequacy and representativeness.

The results of ordinal logistics regression analysis showed that after adjusting for age and BMI, LPW and PFC were positively correlated with K–L grading, and FH was significantly and negatively correlated with K–L grading (Table 4). Multivariable linear regression analysis showed that after adjusting for age and BMI, LPW and PFC were positively correlated with varus deformity, and FH was significantly and negatively correlated with varus deformity (all *P* < 0.05) (Table 5).

**Table 4:** Ordinal logistics regression analysis of K–L grading

	*B*	SE	*P* value	OR (95% CI)
Age	0.108	0.012	< 0.001	1.11 (1.09–1.14)
BMI	0.010	0.028	n.s	1.01 (0.96–1.07)
LPW	0.656	0.057	< 0.001	1.93 (1.72–2.16)
PFC	0.125	0.015	< 0.001	1.13 (1.10–1.17)
FH	-0.632	0.044	< 0.001	0.53 (0.49–0.58)

*K–L grading* Kellgren and Lawrence grading, *LPW* lateral plateau widening, *PFC* proximal fibula curvature, *FH* fibular head height, *B* regression coefficient, *SE* standard error of regression coefficient, *OR* odds ratio, *CI* confidential interval, n.s., *P* > 0.05

**Table 5:** Multivariable linear regression analysis of comprehensive principal component score of varus deformity

	Unstandardized coefficient	Standardized coefficient B	*t* value	*P* value	B 95% CI	
	0.154		0.469	0.639	−0.490	0.798
Age	0.016	0.110	5.423	< 0.001	0.010	0.021
BMI	0.004	0.014	0.681	n.s	−0.008	0.017
LPW	0.174	0.403	16.558	< 0.001	0.153	0.194
PFC	0.017	0.174	7.918	< 0.001	0.013	0.022
FH	−0.131	−0.356	−15.221	< 0.001	−0.148	−0.114

*BMI* body mass index, *LPW* lateral plateau widening, *PFC* proximal fibula curvature, *FH* fibular head height, *B* regression coefficient, *CI* confidential interval; n.s., *P* > 0.05

## Discussion

In radiographic studies of knee OA, the lateral anatomical and morphological characteristics of the knee have not received sufficient attention, possibly because lateral degeneration seems to be less severe than medial degeneration or because the fibula is not the main component of the knee joint and bears little axial load [13, 14]. However, lateral structures play a role in the degeneration of the knee; the bony support of the fibula has a role in the mechanism of knee OA, which was named the “nonuniform settlement” theory [8, 9]. The soft tissues of the lateral knee are important for knee stability. The posterolateral ligament complex, including the lateral collateral, arch, and popliteal ligaments and popliteal tendon, prevents varus deformity and abnormal external rotation of the tibia [15–20]. These morphological abnormalities are obvious in knee OA [21–23]. Therefore, lateral knee structures deserve more attention, especially in knees with OA.

To our knowledge, this is the first large study to describe the anatomy and morphology of the lateral knee joint and its association with knee OA. Our results showed that in varus knee OA, the anatomical position and morphology of the lateral knee on radiography changed, and may be determined by changes in the mechanical environment of the lower limbs. These changes were accompanied by changes in K–L grade and the morphological indexes MPTA, JLCA, and HKAA, suggesting that LPW and PFC are significantly and positively correlated with the severity of knee OA and genu varus deformity, while FH is negatively correlated.

LPW is sensitive to knee alignment and indicates the relative location and distance between the proximal lateral tibia and lateral femoral condyle by drawing two lines perpendicular to the medial tibial articular surface [24]. Studies have used LPW to evaluate lateral tibial plateau fractures [24]. We found that LPW can also be used to evaluate the degree of lateral alignment in patients with varus knee OA. The average LPW of a healthy knee is 0.02 ± 2.03 mm, so the lateral aspect of the tibial plateau should be collinear with the lateral femoral condyle [24, 25]. Here, almost all LPW measurements in patients with varus knee OA were positive and were closely related to knee OA occurrence, progression, and varus deformity. According to past study reports, the relative outward displacement of the tibial plateau, a positive LPW, represents an increase in stress in the lateral structure [26], which also affects the state of the fibula head [24, 25]. In addition to the view of joint mismatch in the previous studies, we believe that the tibial intercondylar ridge is closer to the lateral femoral condyle, resulting in increased structural pressure of the lateral knee structures. So, a change in LPW might be the direct cause of increased stress in the lateral structures when varus OA occurs.

The PFC describes morphological variation in the fibula instead of the fibular shaft axis. Kuroda et al. defined the fibular shaft axis as the line connecting the center of the fibular head to the center of the lateral malleolus; it is commonly used as a radiographic landmark for planning total knee arthroplasty [27]. PFC was defined as the angle formed by the proximal medullary cavity central line and the middle medullary cavity central line of the fibula in a previous study with inaccuracy because the curvature was described by angle [10]. PFC reflects the degree of bending in the proximal fibula with a new definition used here and calculated by the formula mentioned above, and it is proportional to lateral stress. We found that the PFC was significantly correlated with JLCA, MPTA, and HKAA and directly or indirectly affected the lateral structure of the knee, similar to the fibular shaft axis on plain radiographs. Xie et al. reported that the medial cortex of the proximal fibular shaft is a reliable landmark for the mechanical axis of the tibia in positive HKAA knee OA [28]. Our results suggest that the validity of the fibular shaft axis may be an objective manifestation of changes in mechanical stress on the fibula, which are influenced by changes in the load along the tibial mechanical axis. This suggests that changes in the mechanical axis of lower limbs contribute to changes in both the tibia and fibula, rather than just one in isolation. Because of the proximal tibiofibular joint structure, little dislocation of the fibula occurs in the presence of genu varus deformity. The lateral muscles and tendons place more tractive force on the lateral fibular cortex, as demonstrated by the periosteal reaction in the lateral cortex of the fibula on OA radiographs. This mechanism may be the main cause of fibula bending.

We proposed to use the parameter FH to describe the height of the fibular head relative to the tibial plateau. The decrease in FH observed in knee OA indicates that the fibula shifts upward relative to the tibial plateau. FH was significantly and negatively correlated with K–L grade (odds ratio [OR] = 0.53), JLCA, and HKAA, and was significantly and positively correlated with MPTA. According to Preuschoff, the vertical force acting on the fibula is a tensile force, not a compressive force. This explains the upward movement of the fibula, although it may also result from settling of the lateral tibial plateau, which should be examined in further studies. LaPrade et al. reported that the fibula head affects the force and proximal tibiofibular articulation via the lateral structures, which transmit and distribute the lateral tensile force generated by limb gravity [29]. Theoretically, the function of the posterolateral ligament complex declines as the fibula shifts upward because it relaxes. Simultaneously, the pressure in the medial knee compartment increases [30, 31], which eventually leads to knee degeneration, such as hyperosteogeny and osteosclerosis [32]. Consequently, the decreased FH could be a lateral knee characteristic that aggravates knee OA.

In addition, our results showed that there are statistical correlations among the three imaging indexes about lateral knee structure. It is suggested that the three morphological changes in the lateral knee have synchronicity and consistent indicative significance. Referring to the characteristics of medial indexes about varus knee joint degeneration, we collectively refer to the three lateral indexes as indexes about lateral knee joint degeneration. Commonly, degeneration is associated with increased pressure in the knee compartments, which involve medial and lateral compartments to varying degrees. The consistent indicative significance of the three lateral morphological changes could be reflected by that they were all related to increased pressure on the lateral structures of the knee. Theoretically, the abnormal increased pressure, the settlement of the tibial plateau, the O-shaped deformed lower limb and the increased traction on the fibula head together lead to a long-term bowlike pressure state of the fibula, which is regarded as the important mechanism for the fibula shifts upward and the bending of the fibula shaft. As described above, an increased LPW indicates an abnormal increase in pressure on the lateral knee compartment. And, the mechanical environment changes caused by these morphological deformities could in turn lead to the aggravation of degeneration.

The interaction between the fibula and tibia may be involved in adaptive changes in the pathogenesis and progression of knee OA, but this is currently unclear. Lacking superior or inferior bony support, the fibula cannot bear gravity directly but anchors to the tibia via the interosseous membrane and proximal and distal tibiofibular joints. Considering the structural proximity of the tibia and fibula, factors influencing fibula changes in OA might include increased traction of the lateral knee joint and proximal tibia varus deformation on the fibula, which would gradually increase in prominence with knee OA severity. There is extensive evidence to show that changes in the tibia's anatomical and mechanical features affect the fibula's position and morphology, especially the fibula position [33–35]. The traditional view is that the main function of the fibula is to dissipate and transmit axial loads during weight-bearing, but recent studies have suggested mechanical self-adaptability of the fibula in OA and the influence of the fibula on the tibial load in turn [36–38]. Therefore, changes in the anatomical and morphological characteristics of the fibula and tibia in OA may help to explain the mechanical interaction between the tibia and fibula during knee OA.

Generally, varus knee OA involves medial knee compartments first and gradually involves the lateral. When medial and lateral knee compartments are all involved which is the end-stage osteoarthritis, total knee arthroplasty is the major surgical treatment. However, noninvasive treatments, intraarticular puncture and injection, high tibial osteotomy, and unilateral knee arthroplasty are also common ways for knee OA with different severity grades; therefore, it is not enough for traditional indicators to focus only on the degeneration of the medial structures according to the requirements of targeted minimally invasive treatment; and the lack of a method for evaluating degeneration of the lateral knee structures may be an important reason for the lack of standard indications for total knee arthroplasty. Based on the evaluation of the lateral knee degeneration, the classification of knee OA severity will also be more accurate and effective, and this is worthy of further studies. Consequently, although our study focuses on the three morphological changes in the lateral knee, it could provide ideas for enabling a more comprehensive assessment of varus knee OA and for a more reasonable treatment for the knee OA by regarding the medial and lateral compartments as separate structures. Furthermore, it will provide us with ideas for predicting the risk of total knee arthroplasty.

This study has several limitations. First, this study involved a large-sample radiographic analysis, so biomechanical findings were neglected. Imaging variation in the fibula does not directly reflect mechanical changes around the knee joint. Second, the rotational relationship between the tibia and fibula cannot be measured using plain radiography of the knee, although this relationship may exist in knee OA. Third, the impact of rotation or flexion of the limbs on the measurements cannot be observed on 2D radiographs.

## Conclusion

Regular morphological changes, including lateral dislocation of the tibial plateau, proximal fibula bending, and upward movement of the fibular head, take place in the lateral knee with varus knee OA. Changes in LPW, PFC, and FH are significantly correlated with varus knee OA occurrence, and these parameters will enable a more comprehensive assessment of varus knee OA severity and deformity. Furthermore, these adaptive changes in the lateral knee joint could reflect the mechanical synergism and interaction of the tibia and fibula promoting varus deformity in knee OA.

## Data Availability

The final dataset will be available from the corresponding author.

## Abbreviations

OA: Osteoarthritis
LPW: Lateral plateau widening
PFC: Proximal fibula curvature
FH: Fibula height
BMI: Body mass index
K–L grade: Kellgren and Lawrence grade
MPTA: Medial proximal tibial angle
JLCA: Joint line convergence angle
HKAA: Hip–knee–ankle angle
AP: Anteroposterior
